# Engaging With a Participatory Research Team: Evaluation of Involving Older Co‐Researchers With Experience of Falls in the SeFallED Project

**DOI:** 10.1111/hex.70908

**Published:** 2026-10-12

**Authors:** Nadja Reeck, Tim Stuckenschneider, Laura Himmelmann, Georg Lehmann, Margarethe Mammes, Margrit Pape, Bettina Reineking, Tania Zieschang, Anna Levke Brütt

**Affiliations:** ^1^ Department of Health Services Research Carl von Ossietzky University of Oldenburg Oldenburg Germany; ^2^ Member of the Participatory Research Team Oldenburg Germany; ^3^ Department of Medical Psychology University Medical Center Hamburg‐Eppendorf Hamburg Germany

**Keywords:** geriatric, older people, participatory research, patient and public involvement, PPEET, PPI, public and patient engagement evaluation tool

## Abstract

**Introduction:**

There is increasing emphasis on the involvement of older patients in research. A comprehensive and systematic evaluation of this involvement, using established tools, is essential to ensure its effectiveness and impact. This study aims to describe this involvement of older individuals in a geriatric research project and to examine its outcome and impact.

**Methods:**

A participatory research team (PRT) consisting of a heterogeneous group of older adults with experience of falls was set up. The public and patient engagement evaluation tool (PPEET) was used as a means of evaluation. Process documentation was also used for evaluation. All data were analysed descriptively.

**Results:**

Eight older individuals were selected for the PRT, and 29 meetings were held during the 3‐year project period. Due to health‐related absences, three new PRT members were admitted after the first half of the project. The positive atmosphere at the meetings was emphasised. Nevertheless, at the initial stage, there was some ambiguity regarding the purpose. The PRT had an influence on various research phases (e.g., design of the focus groups). All persons involved emphasised the supportive contextual factors, but in the beginning PRT members highlighted the need for more information.

**Conclusion:**

The PRT is a promising strategy for involving older people; however, health‐related absences must be considered. A personal approach is a crucial factor for PRT members (e.g., time in meetings to share own experiences). The PRT had a multifaceted influence on the project, with a heightened emphasis on public relations. The contextual factors (e.g., infrastructure) were supportive, but greater emphasis should be placed on information structures for PRT members in future projects.

**Patient or Public Contribution:**

A PRT consisting of a heterogeneous group of older individuals with a history of falls was involved throughout the entire project duration. All four members who were part of the PRT at the end of the project read and revised this paper.

**Trial Registration:**

German clinical trial identifier: DRKS00025949

## 1 Background

Patient and public involvement (PPI) has become increasingly prominent in health services research and is widely promoted as a means to enhance research quality [1, 2], increase democratic legitimacy [3, 4] and improve the relevance of research to end users [4]. In addition, several research funders now explicitly encourage or require the inclusion of PPI as a condition for funding [5, 6, 7].

The National Institute for Health and Care Research (NIHR) defines ‘involvement in research as research being carried out “with” or “by” members of the public’ [4]. This definition emphasises the active partnership between academic researchers and members of the public, leading to an influence on research [4]. The extent of involvement of members of the public can vary in terms of decision‐making power, for example, from advisory to sole decision‐making power [8]. Within the field of PPI, a range of terms is used, including patient engagement and participatory research, which are often used interchangeably [9]. However, these terms are not universally consistent in their definition [9]. Additionally, the implementation of PPI has so far been inconsistent in Europe, and it is not firmly embedded in healthcare systems [5]. The role of PPI often remains marginal or tokenistic [5]. Further, the members of the public should get involved at all stages of the research cycle, from identifying and prioritising research to evaluating impact [4]. However, the review by Manafo et al. [10] indicates that involvement has mainly been in the early stages of projects and less frequently throughout the duration of the project, highlighting the need for a greater number of projects with PPI at all stages.

There are already reviews regarding the implementation of PPI which focus on the factors that support (e.g., positive relationship and good accessibility) and those that hinder involvement (e.g., lack of role clarity and limited resources) [1, 11, 12]. Previous reviews have also highlighted positive and negative consequences of PPI for academic researchers, co‐researchers (= members of the public) and the research project (e.g., academic researchers gain a better understanding of the research field; overload for co‐researchers; higher costs for the research project) [1, 2, 11, 13]. However, studies with PPI have often lacked systematic evaluation [10, 14], primarily using qualitative methods [15] and focusing on the research process instead of the impact on the research outcome [16]. There are already a number of frameworks (e.g., CUBE) [17] and tools (e.g., public and patient engagement evaluation tool [PPEET] and Patient Engagement in Research Scale [PEIRS‐22]) [18] for evaluating PPI, but the specific evaluation methods are often not set out in the publications [19]. Despite the intrinsic moral and political value of PPI, an evaluation is considered essential in view of its complexity to draw conclusions for good practice [20, 21]. Research is needed to identify approaches that work and their specific effects on outcomes, considering the context [13, 20, 22].

When considering the context, the specific group of people involved in research projects is also relevant. There is an increasing number of studies involving older individuals with health problems such as those living with frailty, multimorbidity or following a stroke, despite the associated challenges (e.g., possible physical and/or cognitive impairment) [1, 11, 23, 24]. However, there is a limited number of studies involving older individuals with health problems of this nature at all stages of the research, and we found no study that additionally used a standardised evaluation tool regarding process and impact on outcome regularly throughout the duration of the project.

The present study describes and evaluates the PPI approach implemented in the research project ‘Sentinel fall presenting to the emergency department’ (SeFallED) [25], which investigates the functional trajectories and needs of older adults following a fall leading to presentation at the emergency department. The SeFallED project is a mixed‐methods study comprising several components that use quantitative methods (comprehensive geriatric assessments at home and in the gait laboratory) and qualitative methods (focus groups) [25]. This manuscript focuses on the PPI approach within the SeFallED project; findings from the other components of the project are reported separately (e.g., focus groups [26] and quantitative analyses [27, 28, 29]). The collaborative work carried out in partnership with individual PRT members in relation to the moderation and analysis of the focus groups has also been published separately, as these specific project tasks involved a higher level of participation and additional evaluations [30]. The SeFallED project involves older individuals with fall experiences in a participatory research team (PRT) and places the implementation of this PPI approach and its evaluation at the centre, regularly utilising the PPEET [31, 32, 33] throughout the project and integrating different perspectives (academic researchers and co‐researchers). A key strength of the PPEET, a standardised quantitative evaluation tool also using free text questions, is its capacity to facilitate descriptive statistical analyses of results and a comparison over time [34]. This study pertains to process evaluation as described in the Medical Research Council (MRC) framework, with *implementation (what is implemented and how is it implemented?)*, *mechanisms of impact (how does it produce change?)* and *context (how does context influence the implementation and outcomes?)* designated as key components enabling a comprehensive evaluation [35]. This framework has been deemed to be a suitable model for the evaluation of PPI in the SeFallED project, as the involvement process can also be regarded as a complex intervention because of the strong influence of the context [36]. The aim of our project was to answer the following research questions:

− How can a PRT consisting of older people with fall experiences be included throughout the entire duration of a geriatric research project? *(implementation)*
− How can the involvement of a PRT produce change? *(mechanisms of impact)*
− How can the context influence the implementation and the outcomes in the geriatric research project? *(context)*

## 2 Methods

This methods section describes how the PPI approach was planned at the start of the project as part of the research proposal and study protocol [25]. Since the specific design of the PPI approach in the SeFallED project depended largely on the context (e.g., the co‐researchers involved) [13, 20, 22, 36] and evolved over the course of the project, its actual implementation and conduct during the project period are described later in the results section. This is also consistent with the research question stated in the background concerning the implementation of the PPI approach, based on the MRC Framework [35].

The present study used a participatory approach which was implemented through the involvement of a PRT throughout the project duration. As outlined in the research proposal, the objective was to establish a PRT comprising four older individuals with a history of falls, who were heterogeneous in terms of gender, age, education, country of birth, housing circumstances and physical limitations. These older people should be involved as co‐researchers (e.g., giving feedback on recruitment strategies and focus group guidelines) to consider the target group's perspective of older individuals with lived experience of falls in our study [25]. The project was scheduled to encompass a total of 30 meetings within the 3‐year funding period. The research proposal included €14,700 for the expenses of the PRT. Furthermore, a significant proportion of the 65% position of a PhD student, which was funded by project resources, involved responsibility for the PRT. This PhD student was primarily responsible for supporting the PRT, which included, amongst other things, organising and running the PRT meetings. Moreover, this PhD student served as a link between the PRT and all university staff involved in the project (= SeFallED project team). The PRT meetings were attended not only by PRT members but also by members of the project team. In addition, there were also regular meetings within the project team without the PRT members. During these meetings, the person responsible for the PRT would, for example, ask the other members of the project team for suggestions on relevant topics for the PRT meetings, or comments made by the PRT and their implementation would be discussed. Figure 1 illustrates the collaboration between the PRT and the project team using PRT meetings as an example.

**Figure 1:**
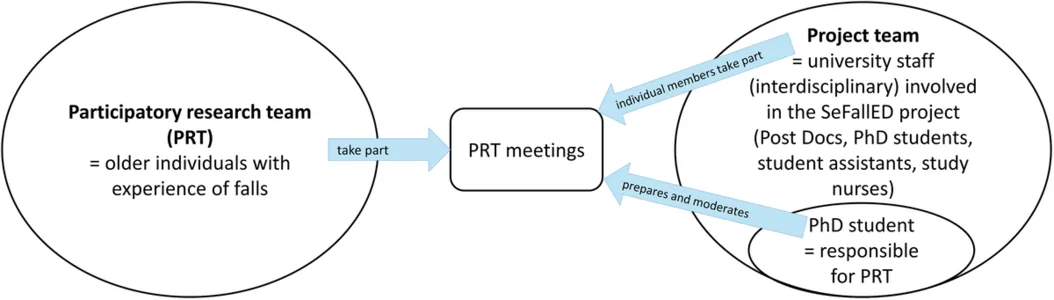
Collaboration between the PRT and the project team (e.g., PRT meetings).

The SeFallED study, as well as the participatory approach, was approved by the Medical Ethics Committee of the University of Oldenburg (2021‐120, 2021‐106). Moreover, the SeFallED study was prospectively registered in the German Clinical Trial Register (DRKS00025949). The *Guidance for Reporting Involvement of Patients and the Public* (GRIPP2) checklist was used as a guide for this publication [37] (see Supporting Information File 1).

### 2.1 Evaluation

#### 2.1.1 Data Collection

The evaluation in this study was based on two parts. A proportion of the data was derived from the PPEET results sheets, with the remainder being drawn from process documentation.

The German translation of the PPEET [31, 33] was used for evaluation as it includes the key components of the MRC framework (*implementation*, *mechanisms of impact*, *context*). Each PPEET questionnaire is aimed at a specific target group, with the Participant Questionnaire intended for patients and citizens and the Project Questionnaire for the engagement implementers (e.g., project leads and staff members) [31]. The Participant Questionnaire consists of four parts (communication and supports, sharing your views and perspectives, impacts and influence, final thoughts). We have selected the Participant Questionnaire Module B for ongoing/long‐term engagement initiatives (Module A = one‐time engagement activities). The Project Questionnaire also consists of different Modules (regardless of the extent of involvement), with Module B encompassing four parts (integrity of design and process; influence and impact; collaboration, common purpose and partnerships; final reflections) and Module C including eight Items (e.g., influence of PPI on project's outcomes). At 6‐month intervals (T1–T5), the PRT members answered the Participant Questionnaire, and the project team filled in the Project Questionnaire Module B (open‐ended questions and Likert‐scale 1 = *strongly disagree* to 5 = *strongly agree*). Additionally, the project team filled in the Project Questionnaire Module C for evaluating the impact of the involvement after 1 and 2 years (= two times [T1 and T2]; open‐ended questions and Likert Scale [Item 1 distinct numbering: 1 = *no influence* to 5 = *strong influence*]). All completed forms were labelled with a personally generated password. All PPEET results were discussed in the subsequent PRT meeting to enable transparency and further reflections [34].

The other part of the evaluation focused on process documentation. This included meeting protocols and PRT's time sheets for working hours, which were particularly significant in terms of evaluating the implementation. Further, all comments made by PRT members, which were recorded in the PRT meeting protocols and in written feedback on tasks between PRT meetings, were systematically documented in a table (see Table 3). The project team also documented whether and how each comment was implemented. If a comment did not lead to any changes in the project, the reason was recorded. These tables were primarily relevant for assessing the mechanisms of impact.

In summary, the data collection methods can be mapped to the various components of the MRC Framework (implementation, mechanisms of impact and context) [35]. The data for the first level of the MRC Framework, implementation, was based primarily on process documentation and individual items in the PPEET Participant Questionnaire. The mechanisms of impact were covered by Module C of the PPEET Project Questionnaire, the PPEET Participant Questionnaire and the process documentation in the form of tables detailing the PRT members' comments and their implementation. The context component was addressed in particular by the Participant Questionnaire and the Project Questionnaire Module B.

#### 2.1.2 Data Analysis

N.R. analysed the results of the PPEET Participant Questionnaire and Project Questionnaire Module B and C regarding frequencies using IBM SPSS Statistics 27 software [38]. All free text answers of the aforementioned questionnaires were analysed qualitatively by N.R., summarising the topics mentioned.

The process documentation (including meeting protocols, PRT's time sheets for working hours and systematic documentation of PRT's comments and their implementation) was analysed descriptively by N.R. For example, using the meeting protocols, the number of PRT meetings and the number of participating PRT members and project team members were counted, and average attendance numbers were calculated. Based on the records of PRT members' working hours, we calculated the additional costs arising from the expense allowances paid to PRT members. The systematic documentation of PRT members' comments and their implementation made it possible to summarise these comments.

## 3 Results

The results were structured according to the key components of the MRC framework (*implementation*, *mechanisms of impact* and *context*) relating to the research questions. The results of the analysis of the PPEET (all ratings of the Participant Questionnaire and Project Questionnaire Module B and C can be seen in Supporting Information Files [Link], [Link], [Link]) and the project documentation were assigned to the matching key component.

### 3.1 Implementation

#### 3.1.1 PRT Members

In July 2021, an article in a local newspaper introduced the beginning of the SeFallED project and included a call for older individuals to join the PRT. As a result, 56 interested persons were included on a list which contained contact details as well as sociodemographic information (sex, age, education, country of birth, housing situation, use of mobility assistive devices and use of outpatient care services), the person's experience of falls and expectations regarding involvement in the PRT. From this list and considering sociodemographic diversity, the project team invited eight individuals to an introductory meeting in September 2021 (see Table 1). In light of the considerable interest expressed and the project team's reservations regarding the long‐term participation of all interested individuals within the PRT, the initial plan of four PRT members was revised, with the number being increased to 8. The introductory meeting focused on getting to know one another. For example, rules for collaboration (such as respectful communication and a safe space) were discussed, everyone introduced themselves, expectations were shared, and the SeFallED project was presented. There was no explicit training on research content for the PRT members. Instead, the necessary information was provided as and when required during the course of the project's various work packages. Several training sessions were held for the relevant PRT members only in the context of the collaborative work on the focus groups [30]. In addition, training sessions were organised for individual PRT members on how to take part in video conferences, as some PRT meetings had to be held online due to the coronavirus pandemic and some PRT members had never taken part in a video conference before. Shortly after the introductory meeting, the number of PRT members was reduced to 6 due to health‐related cancellations.

**Table 1:** Characteristics of PRT members.

	PRT members
*N*	11
Sex	
Female	6
Male	5
Age upon joining PRT	
Mean (range)	74.9 (63–88)
Highest school graduation	
First general education leaving certificate after Grade 9	5
General education leaving certificate after Grade 10	1
Higher education entrance qualification	5
Country of birth	
Germany	10
Poland	1
Housing situation^a^	
Living alone	2
Living with partner	8
Living with family	1
Multigenerational house	1
Use of mobility assistive devices	
None	6
Walking stick	3
Walker	2
Period in the PRT	
Whole project duration	1
Since October 2022 until end	3
Until December 2023	1
Until October 2023	1
Until January 2023	1
Until November 2022	1
Until September 2022	1
Until November 2021	1
Invited to introductory meeting	1

^a^ One PRT member had two residences.

As the project progressed, there were drop‐outs due to health‐related issues (see Figure 2). In the free text responses of the PPEET Participant Questionnaire, the PRT members addressed this issue, given that the group size was deemed to be too small. In autumn 2022, it was jointly decided to expand the PRT, which was described as positive in subsequent evaluations. In this context, the aforementioned list proved to be helpful, and three additional persons joined the PRT. By the end of the SeFallED project, there were four members regularly participating in PRT meetings. In summary, a total of 11 older adults were involved as PRT members throughout the project duration. It should be noted that all PRT members who withdrew from the PRT were informed that they were welcome to rejoin at any time, although none have done so.

**Figure 2:**
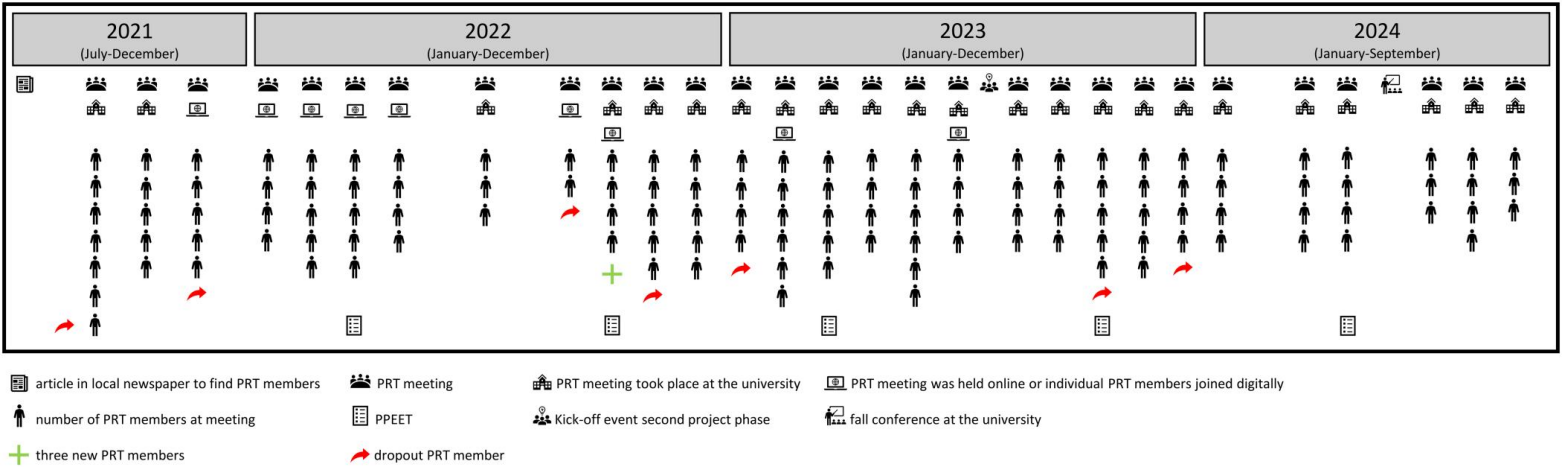
Overview of PRT meetings.

#### 3.1.2 Organisational Aspects

The PRT was involved in the SeFallED project from September 2021 until the end of the project in September 2024. A total of 29 PRT meetings were held, attended by 2–7 PRT members (*M* = 4) and 3–5 academic researchers and study assistants (*M* = 4). Twenty meetings took place at the university. Six meetings were organised online and three as hybrid events (see Figure 2).

N.R. organised 2‐h PRT meetings approximately monthly on a Thursday morning with complimentary drinks and snacks. N.R. was the main moderator of the meetings. Besides the PRT members, other project team members took part.

In addition to these meetings, the PRT members also completed individual tasks in between. These were tasks carried out on their own (e.g., commenting on consent forms) or additional individual appointments with project staff, such as piloting the tests in the gait laboratory. Moreover, the PRT members had the opportunity to attend congresses and workshops.

A total of 596 working hours, including time spent at PRT meetings and on individual tasks, were recorded by the PRT members on time sheets. Accordingly, a total of €11,920 in expense allowances (€20 per hour) was paid to them. Additional costs to the sum of €222 were incurred as a result of one PRT member attending a congress. Further, there were minor costs for snacks and drinks at PRT meetings.

#### 3.1.3 Working Method

Every PRT meeting started with a welcome and short exchange about the current situation and subsequent thoughts on the previous meeting (including protocol) from every participant. Further, all participants were asked if there were additional points for the meeting agenda. This was followed by substantive agenda items (e.g., discussion about the recruitment process), including a short break. Each meeting ended with a conclusion and reflection on the meeting from all participants. A detailed written record was kept during each meeting and a summary sent to the PRT members. All topics involving the PRT can be seen in Table 2. For example, PRT members read and commented on the consent forms for the quantitative and qualitative data collection, discussed the recruitment strategy with the project team, tested and provided feedback on the assessments used during the home visits and gait laboratory tests, developed the focus group interview guide with the project team, read and commented on the newsletter for the contact database, created a poster about the PRT for a conference at the university, organised a kick‐off event for the second project phase with the project team and gave feedback on the planning of the second project phase [39].

**Table 2:** Topics in the PRT meetings and tasks between meetings.

Introductory meeting	Getting to know each other
	Expectations of involvement
	Project introduction
Designing and managing	Consent forms for quantitative and qualitative data collection
	Flyer emergency department and focus groups
	Recruitment in the emergency department
	Monthly calls to the study participants
	Testing the home visits
	Piloting of the gait laboratory tests
	Results forms for study participants
	Christmas letters for study participants
	Planning focus groups
	Development of guidelines for focus groups
Undertaking	Co‐moderation of focus groups
	Co‐analysis of focus groups
Disseminating	Public relations work (e.g., newspaper articles)
	Newsletters contact database
	Preparations for conference at the university (e.g., poster creation)
	Presentation and discussion of project results
Planning second project phase	Kick‐off event second project phase (invitations, planning and realisation of the event)
	Planning second project phase (e.g., organisation of the PRT work)

The PRT had a primary advisory role in the SeFallED project. This means that the PRT discussed with the project team, but the project team was empowered to decide. However, there were some research tasks where PRT members collaborated on a partnership basis, with decision‐making power shared with the project team (e.g., focus group analysis [26, 30]). In order to facilitate transparency with regard to the decisions made in the course of the project, N.R. systematically documented all PRT comments in a table (see Table 3) and presented these to the PRT, together with the reasons why comments were possibly not implemented. In addition to providing feedback on existing drafts, which was documented in these tables, the PRT was occasionally directly involved in the development of study documents that had not yet been drafted by the project team, such as the guidelines for focus groups.

**Table 3:** Systematic documentation of comments by PRT members and their implementation in the project (examples).

Procedure for (monthly) telephone calls with study participants		
Comment by PRT member(s)	Implementation (yes/no)	Explanation
Provide acute support to make subjects feel better	No	Given the observational nature of the study, it is not feasible to provide acute support.
Self‐advocates should initiate telephone communications	No	The current data protection regulations, in conjunction with the substantial volume of telephone calls and the coordination thereof, present a considerable challenge in facilitating calls from those affected, for example, PRT members.
Sometimes there are very mundane reasons why you don't want to take part, e.g., I can't tidy up my home on my own after the fall and therefore don't want anyone to come to my home	Yes	In the event of any concerns, testing at the university was always offered in the past. In the current structure, this option is explicitly mentioned during the procedure, irrespective of any explicit request.
Fixed appointments for telephone calls	No	In the process of appointment scheduling, the preferred times of test subjects are given due consideration (e.g., day care, early or late). However, it is not feasible to make specific appointments, as the scheduling team requires a certain degree of flexibility due to the high number of changes that are made to the schedule.
Instead of ‘How are you doing?’ ask ‘How have you managed so far and how will you manage now?’	Yes	Instead of asking ‘How are you’, the first contact asks ‘How are you coping and how can you cope in the future?’

Guidelines for focus group		
Comment by PRT	Implementation (yes/no)	Explanation
The question: ‘Are you also afraid that the offer will make things worse?’ did not feel like a good fit, was difficult to answer, tended to stop the flow	Yes	The question has been posed in a more open manner.
Guide the introduction round a little more and be more specific about what is reported (otherwise the stories may be too long)	Yes	Specific questions to name and way to university
‘What do you do to keep yourself mobile’ is an unfavourable question; some people may not be doing anything at the moment. Better as a question ‘Do you already do anything to keep yourself mobile?’	Yes	Question adapted
Do not ask for the ‘perfect intervention’, but ‘what is important to you about an intervention?’	Yes	Question adapted

The responsibility for the PRT was transferred to N.R., who was supervised by A.L.B. The position held by N.R. functioned as a conduit between the PRT and the project team of academic researchers and study assistants. The responsibility for communicating with the PRT members and preparing documents for the PRT meetings fell primarily to N.R. Furthermore, she was tasked with the responsibility of processing the comments from the PRT, ensuring their subsequent utilisation by the project team. Consequently, the project team furnished N.R. with feedback concerning the implementation of the aforementioned comments, which was also presented transparently to the PRT. In particular, there was a continuous intensive exchange with T.S. and L.H. in regular team meetings on a 2‐week basis. In these, PRT meetings were planned in conjunction in order to include topics that were currently relevant to the project. Furthermore, direct exchange between the PRT and the project team was also occurring, for example in the form of presentations at PRT meetings and pre‐tests in the gait laboratory.

#### 3.1.4 Feedback

In the free text answers of the Participant Questionnaire, PRT members repeatedly emphasised the positive atmosphere (e.g., open, appreciative and responsive to wishes) as supportive: ‘I was given the feeling that you are part of the project’ (PPEET, Participant Questionnaire, T3). The good guidance (e.g., for conducting video conferences) and the communication between meetings with good accessibility via telephone and e‐mail were perceived as positive. PRT members highlighted the continuity of those involved as an advantage, as it created a sense of familiarity.

The working processes of the PRT were also described as a strength in the free text responses, with the high level of motivation of all PRT members being emphasised. The PRT members underlined the intensive and serious work on specific topics in the meetings and on other tasks, and the good agreement procedures. However, there was also some critical feedback that PRT members sometimes made too digressive contributions and sometimes too much focus was placed on individual words (e.g., Christmas letter). Additionally, in the first PPEET evaluation, one PRT member described, ‘By chance, I ended up in the PRT and would now prefer to be a test subject’ (PPEET, Participant Questionnaire, T1). Another critical point after 6 months was the clarity of purpose for the PRT members, with 67% (four out of six) of them agreeing or strongly agreeing with this item.

### 3.2 Mechanisms of Impact

The impact on outcome was primarily assessed in Module C of the Project Questionnaire from the perspective of the project team and was supplemented by a few items from the Participant Questionnaire. The ratings for all items can be seen in Supporting Information Files 2 and 4 and Figure 3.

**Figure 3:**
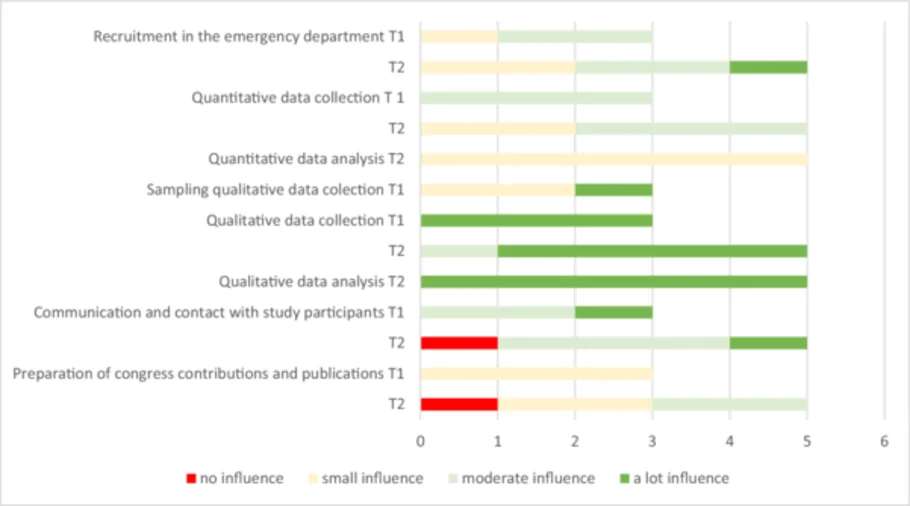
Influence of PRT (PPEET Project Questionnaire Module C). Only the project tasks that were relevant at the time were evaluated, meaning that some aspects were only evaluated at one point in time (e.g., the qualitative data analysis had not yet begun at the time of the first measurement). The response rate at each point in time was 100%, although the participants varied depending on changes in the project team.

All project team members agreed or strongly agreed that the results from the PRT were taken into account, which is supported by the systematic documentation of PRT's comments and their implementation (see Table 3). It is noteworthy that 67% (two out of three) of the project team initially agreed on PRT's influence on the course of the project. However, this perspective shifted towards 100% (five out of five) agreement after 2 years. This finding was analogous to the ratings of the PRT in the PPEET Participant Questionnaire regarding the item “PRT makes a difference” (see Supporting Information File 2), where agreement also increased over time (67% (four out of six) agreed or strongly agreed after 6 months and 100% (six out of six) agreed or strongly agreed after one and a half years) and their free text answers. First, the focus was on ensuring that comments were taken into account and on some critical feedback that the overall impact was considered to be rather small: ‘Not much, as I'm not that knowledgeable!’ (PPEET, Participant Questionnaire, T1). Later on, PRT members identified many specific areas in which they had an impact (e.g., data collection and communication with study participants). Further, they emphasised their diverse life perspectives as older people and knowledge of existing regional structures as strengths in influencing the project.

The Project Questionnaire Module C asked the project team to rate the influence of the PRT in relation to different aspects of the project (see Figure 3). Of the academic researchers, 100% (three out of three) perceived a lot of influence on qualitative data collection after 1 year (T1, T2 = 80% (four out of five)) and qualitative data analysis after 2 years (T2, five out of five). There was a moderate influence seen in the recruitment in the emergency department (T1 = 67% (two out of three), T2 = 40% (two out of five)), in the quantitative data collection (T1 = 100% (three out of three), T2 = 60% (three out of five)), and in the communication and contact with study participants (T1 = 67% (two out of three), T2 = 60% (three out of five)). The majority reported a small influence on the sampling for qualitative data collection (T1 = 67% (two out of three)) and on the quantitative data analysis (T1 = 100% (five out of five)). The influence on the preparation of congress contributions and publications was rated as small at T1 (100% (three out of three)), but increased at T2 to a small to moderate influence (40% (2 out of five) small, 40% (two out of five) moderate).

In free text responses (Project Questionnaire Module C), the academic researchers stated that the PRT's recommendations had been implemented in many cases. However, it was sometimes not possible to implement comments due to limited time and financial resources. The academic researchers emphasised an influence on content, wording and layout at the various research stages, which could also be seen in the systematic documentation of the PRT's comments (see Table 3). For example, the PRT's feedback led to the study participants receiving feedback on their test results if they were interested. Additionally, the PRT members placed a stronger focus on public relations work (e.g., articles in local newspapers), which was highlighted both by the academic researchers ‘The PRT members constantly encourage us as a project team to produce publications that are read by older people in [city] and the surrounding area’ (PPEET, Project Questionnaire Module C, T2) and the PRT members. Moreover, both described a strong impact on the development of the second project phase [39]. PRT members had, for example, a key role in organising the launch event for interested study participants and falls prevention experts. In the course of the event, the current status of the study was presented, followed by a discussion at various World Café tables regarding falls prevention interventions already in place and those that were required. PRT members participated in many tasks such as developing the content and organisational structure of the event, selecting the people to be invited and moderating World Café tables.

Besides the impact on the research outcome, further influences could be assessed. In relation to the impact on co‐researchers, the PRT members emphasised the exchange among each other in the free text responses in the PPEET Participant Questionnaire, which allows them to take away suggestions for their own everyday lives. They also reported learning things such as taking part in a video conference. However, the Participant Questionnaire also included the item to be better informed about health care after a fall and falls prevention, which received lower ratings, with 50%–60% of PRT members neither agreeing nor disagreeing or disagreeing at the different time points (T1: three out of six, T2: three out of five, T3: three out of six, T4: three out of five and T5: two out of four).

In addition, the academic researchers reported in the free text responses (Project Questionnaire Module C) an impact on them as academic researchers, as they gained a better understanding of the target group's perspective, becoming ‘more sensitive to the needs and perspectives of the target group’ (PPEET, Project Questionnaire Module C, T2). They also demonstrated an increased level of self‐questioning and felt more confident and motivated due to PRT feedback.

However, the academic researchers also reported challenging influences on their work. The large amount of time required for PPI was emphasised. The feedback loops resulted in prolonged coordination of decisions, consequently leading to an increase in time pressure. A high level of organisation and communication was required (e.g., prioritising which issues should be discussed). Overall, the academic researchers described in the PPEET free text responses a need for a high degree of flexibility in order to adapt plans on an ongoing basis.

Moreover, academic researchers reported that they had been able to use contacts in the PRT for further activities, but also that colleagues had asked PRT members to help with other projects. The participatory component of the SeFallED project attracted the interest of colleagues and was seen as a model. The added value of the involvement of a PRT could be highlighted, and ‘working with the PRT has also changed the way we work in our organisation beyond the scope of the project’ (PPEET, Project Questionnaire Module C, T2). However, it was also reported that the impact on the project could not always be quantified, which led to the approach being questioned by others.

### 3.3 Context

Three PPEET items focused specifically on the context. In these items, most of the PRT members (Participant Questionnaire) and the project team (Project Questionnaire Module B) strongly agreed that the necessary support was available (60%–100% of the PRT (T1: five out of six, T2: three out of five, T3: five out of six, T4: four out of five and T5: four out of four), 67%–100% of the project team (T2: two out of three, T3: four out of four, T4: four out of five, T5: five out of five; exception T1: one out of four strongly agreed and two out of four agreed)). The project team agreed or strongly agreed that enough time was planned for the participatory approach (between 75% and 100%, T1: four out of four, T2: three out of three, T3: three out of four, T4: five out of five, T5: five out of five).

In the free text answers of the Participant Questionnaire, PRT members described the timing of the meetings as positive, as it provided sufficient time to express their own views. The good catering at the meetings was also mentioned as a supporting aspect. In addition, they emphasised aspects of the infrastructure, such as support with travel costs and good parking facilities, as favourable.

PRT members liked doing tasks at home between meetings to stay on topic. In addition, one evaluation critically noted that more information was desired before the meetings in order to be able to prepare better. As a result, all PRT members were sent preparation materials before the meetings, which were highlighted as positive in a subsequent evaluation. Furthermore, PRT members noted that they would like to have more information on the current status of the project and on the achievement of its goals. This aspect was thus addressed at the beginning of every following meeting.

## 4 Discussion

### 4.1 Implementation

The PPI approach that was implemented in the SeFallED project demonstrated that older individuals with fall experiences can actively engage in a variety of activities over the course of a health services research project addressing the needs and care of older adults after a fall. It highlights organisational procedures as well as the way of working in a PRT, which have been evaluated positively and may serve as a useful reference for future projects involving older co‐researchers with health issues. The high level of interest in participating in the PRT, as evidenced by the significant number of responses to the newspaper article, is indicative of the profound interest among older individuals in becoming involved in research.

Through the evaluation of the PPI approach implemented in the SeFallED project, we have learnt that participants' responsibilities should be clearly defined to determine whether they are providing advisory services or decision‐making responsibilities. In reviews, a lack of role clarity has been identified as a barrier to involvement, whereas clearly defined roles have been described as a supporting factor [1, 12]. For example, in a study included in the review by Ludwig et al. [1], academic researchers and co‐researchers working collaboratively on a cancer research project developed recommendations for PPI through focus groups and subsequent refinement [40]. Among these recommendations, they emphasised the importance of transparent communication about the roles and responsibilities of all those involved [40]. In our SeFallED project, we elected to follow the advisory approach in terms of the extent to which involvement would be pursued, which our PRT members welcomed as it reduced their responsibility and eased the pressure on them. As the project progressed, all those involved received greater clarity regarding the roles of PRT members, facilitating the integration of new members. In this context, transparency around decisions is crucial, making documentation like our tables for PRT's comments and their implementation key tools.

From the perspective of the project team and the PRT, the high number of health‐related absences of PRT members posed a challenge. In the review by Ludwig et al. [1], the health status of co‐researchers is also mentioned as a barrier to involvement. It is, therefore, important to emphasise the commitment required by participation from the outset and to reflect together on whether health problems could prevent continuous involvement, as maintaining the cohesion and continuity of the group is considered paramount. Furthermore, a proportion of unexpected cancellations should also be planned for, and a larger group should be formed. A waiting list, such as our contact database, can also be created to easily find new members for the project, if needed.

PRT members have highlighted the launch event for the second SeFallED project phase [39] as very important, emphasising the high level of involvement as particularly positive. This suggests a desire for substantial involvement and the performance of specific tasks with a tangible outcome, a view also expressed in the wish to be more involved from co‐researchers in the field of stroke survivors in a focus group study by Harrison and Palmer [41], a study included in the review by Baldwin et al. [11]. Preparing this launch event was just one example among many of PRT members collaborating with the project team to develop ideas in contrast to discussing drafts developed by the project team. It was asserted by PRT members that both approaches are relevant and that the possibility of developing ideas in addition to discussing drafts is of value. The choice between these approaches depends on factors such as member preferences, experiences, task specifics (e.g., less relevant documents may be better discussed with a draft, such as Christmas letters), time available and team size (collaborative development with many people needs more time).

In addition to the substantive work on the SeFallED project, PRT members emphasised the importance of the relational and interpersonal aspects of PRT meetings. Accordingly, beginning and concluding meetings with periods of reflection and providing space for storytelling were considered important components that should not be overlooked. PRT meetings may, therefore, require a different format from meetings involving academic researchers only, with a less tightly structured schedule, sufficient time for informal exchange and the provision of refreshments. This is particularly relevant because the PPI approach adopted in the SeFallED project highlighted the importance of supportive relationships and a positive atmosphere. This finding is consistent with other studies [1, 11, 12, 23] which have identified trust, openness and respect as key characteristics of successful involvement and emphasised the need to allow sufficient time for these relationships to develop. For example, Harrison and Palmer [41] conducted qualitative interviews with stroke survivors and two carers involved in various PPI groups and described supportive relationships as an important factor facilitating involvement. In the SeFallED project, the positive atmosphere was further supported by face‐to‐face meetings at the university, which all participants regarded as beneficial compared with online meetings. Nevertheless, participants were also satisfied with the online format and identified advantages in certain circumstances, including avoiding the risk of coronavirus infection and enabling participation when illness or mobility limitations made attendance in person difficult.

Figure 4 gives an overview of the enablers and barriers for the implementation of the PRT in the SeFallED project.

**Figure 4:**
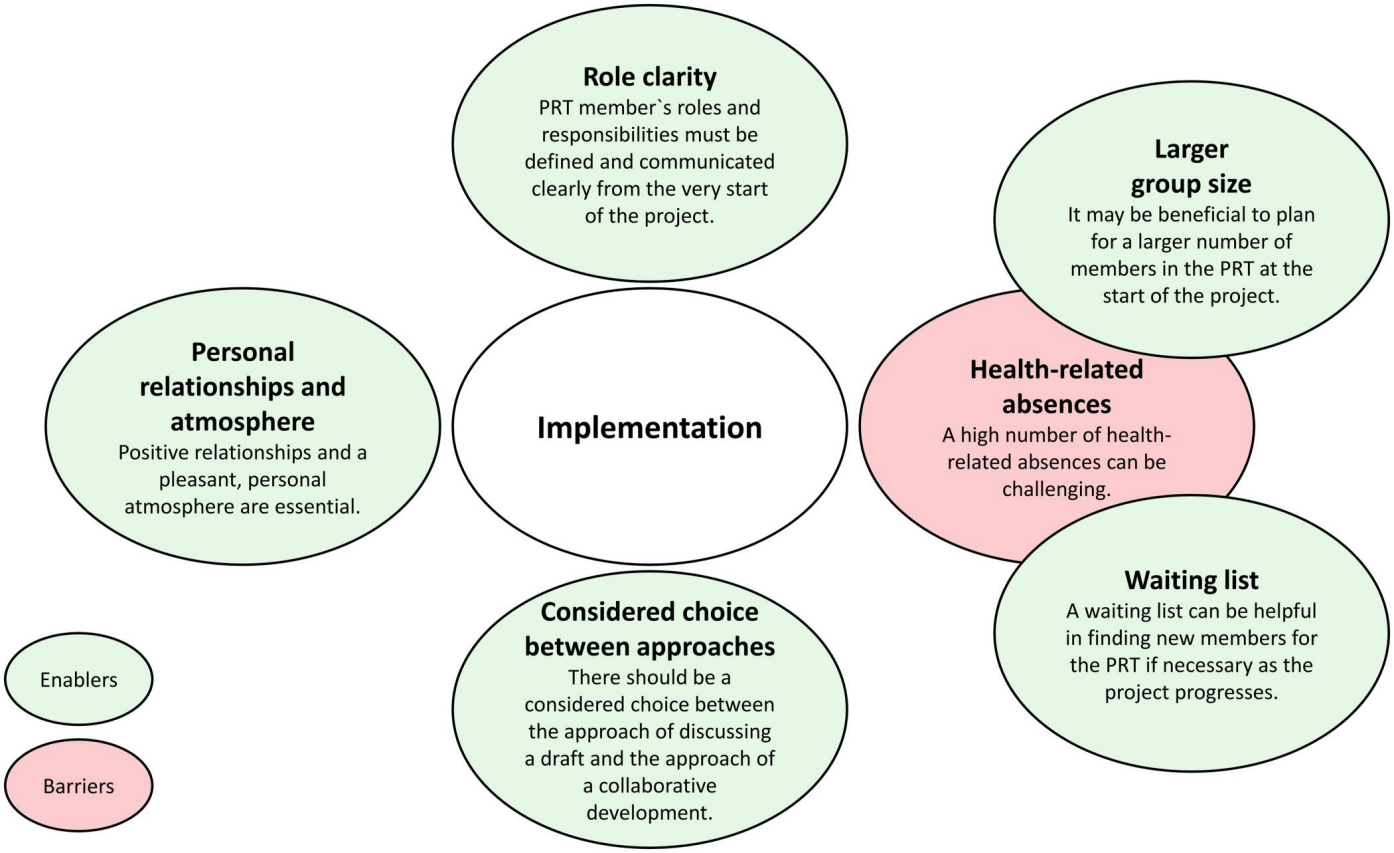
Enablers and barriers for implementation of PPI in the SeFallED project.

### 4.2 Mechanisms of Impact

A notable observation derived from the PPEET results was that both the project team and the PRT recognised a heightened impact of the PRT on the project the longer they were involved. This can be seen to some extent as a process for establishing collaboration, which may also have been promoted by certain prejudices on both sides, which are also described in the review by Ludwig et al. [1] (e.g., scepticism regarding the influence of PRT members without research experience). In this context, PRT members highlighted the aforementioned transparency as particularly important to show PRT's influence. Moreover, they emphasised a specific person who can be contacted with any queries as relevant to the support provided in the event of uncertainties. Other studies also report influences of PPI on the research project [1, 2, 13].

The project team and the PRT both described the strong focus on public relations as a particular influence, which is also briefly mentioned in the review by Baldwin et al. [11]. The PRT focused more on citizens as a target group, resulting in several articles in the local newspaper and the launch event with citizens for the second project phase. However, the PRT had aspired to undertake further initiatives in this direction (e.g., local brochures for older people), but there was not enough time. Such unfulfilled aspirations on the part of the PRT have the potential to engender conflict. However, this was not the case in our instance, presumably due to the positive nature of our relationship and the transparency of our procedures.

The project team noticed the least influence in the quantitative analysis and publications/conference contributions. As part of the quantitative analyses, fixed standardised tests were used that could not be meaningfully adopted by the PRT, resulting in the values calculated by academic researchers primarily being discussed with the PRT at the end, a standpoint that aligns with that of Hannigan [42] and Goulao et al. [43]. With regard to the minimal influence on publications, it should be noted that these were mainly produced after the end of the evaluations, but contact with the PRT was maintained to discuss them, as we see involvement in publications, like others [23], as an important aspect enabling co‐authorships.

The results of the PPEET have suggested that involvement in the PRT has not resulted in members becoming better informed about care after a fall and falls prevention. However, in a discussion meeting in preparation for this publication, PRT members emphasised that, in retrospect, they would assess this differently now. They highlighted an indirect influence, and that they had become more involved with the topic, though no knowledge was directly transferred. They were encouraged to reflect on themselves through statements of other PRT members, which they described as important. The importance of learning was further emphasised by the high value placed on attending scientific conferences, which were regarded as valuable opportunities for expanding one's knowledge. Previous reviews have also reported the positive impact of PPI on the learning of co‐researchers [1, 11].

Moreover, the aspect of learning encompasses not only the acquisition of theoretical knowledge and ideas, but also the accumulation of practical experience. PRT members emphasised the opportunity to utilise devices used in the study (particularly the perturbation treadmill) and thereby gain first‐hand experience as beneficial. Nonetheless, the PRT members also placed considerable value on their interactions with the project team comprising younger individuals, given the rarity and limited scope of their daily interactions with people from this age group. Potential benefits of intergenerational projects have been shown [44, 45], but the majority of studies focus on children rather than younger adults collaborating with older adults. From the perspective of PRT members, it is of particular importance to value and communicate all these possible benefits for the co‐researchers.

### 4.3 Context

Previous studies have highlighted the importance of contextual factors that support older co‐researchers [1, 23]. For example, Markle‐Reid et al. [23] emphasised in their study with older people with multimorbidity that contextual factors, such as those relating to location, must be taken into account because they may hinder participation in PPI research approaches. Within the PPI approach of the SeFallED project, we tried to consider many possibilities to support older co‐researchers (e.g., transport costs, expense allowances, location), which seems to have been successful based on positive feedback from PRT members.

However, with regard to the provision of information materials, it became evident during the course of the project that the level of support required exceeded initial expectations. This was also evident in the PPEET results, which demonstrated that the objectives of the project (including the aim of the involvement of the PRT) were not yet entirely clear to the PRT members. This finding aligns with the study by Markle‐Reid et al. [23] highlighting the potential for misinterpretation of expectations by co‐researchers arising from the distinction between roles as a co‐researcher or a participant in research. It is evident that preparatory documents and the communication of the current project status as fixed agenda items in PRT meetings appear to be crucial support factors in this context.

### 4.4 Strengths and Limitations

The present study makes a contribution to the reporting and evaluation of PPI of older people using the key elements of the MRC framework, which has seldom been done in previous studies. It underscores the efficacy of continuous evaluation in facilitating the implementation of involvement structures. The study demonstrated that this can engender minor adjustments, thereby enabling improvements in the involvement process for the entire project duration. Nevertheless, it is imperative to acknowledge the subjective nature of our evaluation, informed by the perceptions of the individuals involved in the project. This subjective component has the capacity to exert an influence on the outcomes of the evaluation process. In addition, all individuals who completed the evaluation forms were involved in the process, and there was no external evaluator.

The PPEET, a standardised evaluation tool, was used for the evaluation, whereas previous studies have rarely used standardised evaluation tools [19]. One of the PPEET's strengths is that it is a quantitative evaluation tool which also includes open‐ended free‐text questions [34]. The PPEET thus enables descriptive analyses and comparisons over time [34]. However, critical aspects of the PPEET must also be taken into account, such as the limited scope for expressing ideas [34], the failure to identify the most important aspects for those involved [34] and the limited involvement of patients and members of the public in the development of the tool [33].

A notable strength of this study is the comprehensive involvement of the PRT over the course of the project, a feature that has been lacking in previous studies [10]. However, the establishment of our PRT coincided with the start of the funding period. It is evident that the PRT was not involved in the composition of the proposal. The coordination and financing of PPI initiatives prior to the project's funding period remains a challenge [46]. Notwithstanding, in preparing the proposal for follow‐up funding, there was the possibility of the PRT being involved.

As previously outlined, the PRT members demonstrated heterogeneity with regard to several sociodemographic characteristics (e.g., sex, age and education). However, the objective of reaching persons with a migration background was not achieved, which aligns with previous studies reporting difficulties in assembling diverse groups [23, 47]. Beyond the sociodemographic characteristics of a PRT, it is imperative to acknowledge the influence of individual personalities on involvement processes.

## 5 Conclusion

The implementation of a PRT enabled successful involvement of older individuals throughout the course of the SeFallED project. However, health‐related absences posed a challenge, which should be considered when forming a PRT with older individuals. A particular role in the collaboration with a PRT is influenced by a personal approach, for which adequate opportunities should be provided at PRT meetings (e.g., exchange of personal experiences).

Our evaluation showed that the involvement of the PRT exerted an influence on the project (e.g., on study materials), a fact that was particularly evident in the course of the collaboration. For this reason, it is imperative to acknowledge that a period of adjustment is necessary for all involved to establish their roles and for the various influences on the project to become apparent. The involvement of a PRT in research projects can support public relations work and proposals for follow‐up funding. Furthermore, the projects can yield benefits for both academic researchers and co‐researchers, including intergenerational collaboration and valuable insights into research projects.

The contextual factors (e.g., infrastructure) that framed the project provided a favourable opportunity to collaborate with older people. The requirements of PRT members for information regarding the research project and PRT meetings exceeded our initial expectations. Particular emphasis should be placed on the establishment of structures for this in future projects.

## Author Contributions

**Nadja Reeck:** conceptualisation, methodology, data curation, investigation, validation, formal analysis, visualisation, writing – original draft, writing – review and editing. **Tim Stuckenschneider:** data curation, investigation, validation, project administration, writing – review and editing. **Laura Himmelmann:** investigation, validation, writing – review and editing. **Georg Lehmann:** investigation, validation, writing – review and editing. **Margarethe Mammes:** investigation, validation, writing – review and editing. **Margrit Pape:** investigation, validation, writing – review and editing. **Bettina Reineking:** investigation, validation, writing – review and editing. **Tania Zieschang:** data curation, supervision, funding acquisition, project administration, writing – review and editing. **Anna Levke Brütt:** conceptualisation, methodology, data curation, investigation, validation, formal analysis, supervision, funding acquisition, project administration, writing – review and editing.

## Ethics Statement

The SeFallED project including involvement of co‐researchers was performed in line with the principles of the Declaration of Helsinki and was approved by the Medical Ethics Committee of the University of Oldenburg (2021‐120, 2021‐106).

## Conflicts of Interest

The authors declare no conflicts of interest.

## Supporting information


Supporting File 1

Supporting File 2

Supporting File 3

Supporting File 4

## Data Availability

The data that support the findings of this study are available on request from the corresponding author. The data are not publicly available due to privacy restrictions.
