# Supplementary material for: Engaging With a Participatory Research Team: Evaluation of Involving Older Co‐Researchers With Experience of Falls in the SeFallED Project
Source: Health Expect. 2026 Oct 12;29(5):e70908. doi: 10.1111/hex.70908 (PMC13655765; doi:10.1111/hex.70908)
Supplement: Supplementary file 3 [file HEX-29-e70908-s003.pdf]

Additional File 4: Quantitative results PPEET Project Questionnaire Module C - academic researchers

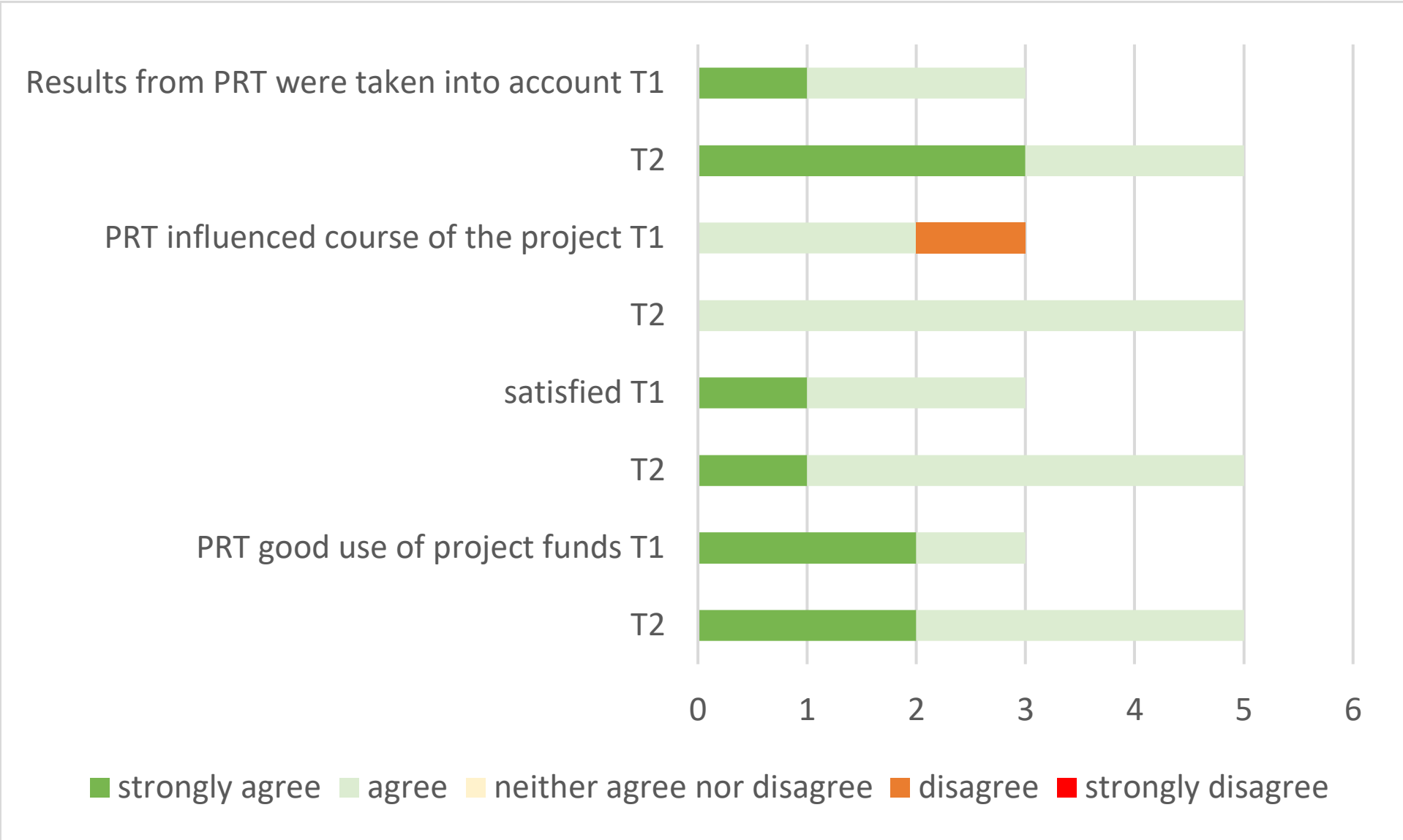

* The response rate at each point in time was 100%, although the participants varied depending on changes in the project team.
